# The Ethanolic Extract of *Lycium ruthenicum* Ameliorates Age-Related Physiological Damage in Mice

**DOI:** 10.3390/molecules28227615

**Published:** 2023-11-15

**Authors:** Boya Cui, Lanying Liu, Tao Shi, Min Yin, Xu Feng, Yu Shan

**Affiliations:** 1 Jiangsu Key Laboratory for the Research and Utilization of Plant Resources, Institute of Botany, Jiangsu Province and Chinese Academy of Sciences, Nanjing Botanical Garden, Memorial Sun Yat-Sen, Nanjing 210014, China; boya_cui@126.com (B.C.); stone981201@163.com (T.S.); epmin@sohu.com (M.Y.); fengxu@cnbg.net (X.F.); 2 National Wolfberry Engineering Research Center, Institute of Wolfberry Engineering Technology, Ningxia Academy of Agriculture and Forestry Sciences, Yinchuan 750002, China; syndia1980@126.com

**Keywords:** anthocyanins, spermidines, oxidative stress, cognitive impairment, cellular senescence

## Abstract

Aging and age-related diseases are important study topics due to their associations with progressive physiological damage to genes, cells, tissues, and the entire organism, which ultimately affects the functional efficiency of organs. *Lycium ruthenicum* Murr. is a functional food that is known for its high contents of anthocyanins and spermidines, both of which have been demonstrated to have positive effects on anti-aging activity and anti-oxidation. In this study, we used HPLC-MS to analyze the constituents of *L. ruthenicum* Murr. Extract (LRM) and investigated their potential mechanism for exerting antioxidative effects in D-galactose (D-Gal) aging model mice. LRM (25 mg/kg, 50 mg/kg, and 100 mg/kg) improved cognitive function in D-Gal-treated mice, as shown by reduced escape latencies and increased platform crossings in behavioral tests. We measured the contents of lipid peroxidation (LPO) and malondialdehyde (MDA) and the enzyme activities of the antioxidant enzymes superoxide dismutase (SOD) and glutathione peroxidase (GSH-Px) in mice serum and brain after 6 weeks of D-Gal treatment. LRM decreased the contents of LPO and MDA and increased the enzyme activities of SOD and GSH-Px, indicating the protection effect of LRM against D-Gal-induced oxidative stress. Additionally, LRM can inhibit oxidative stress in cells by reducing intracellular ROS levels and restoring mitochondrial membrane potential, thereby inhibiting paraquat (PQ)-induced cellular senescence and delaying cell aging. Therefore, LRM has the potential to be a healthcare product for the treatment of age-related diseases.

## 1. Introduction

Aging is a complex process that affects the body at different levels. These changes make the body more vulnerable to various diseases and eventually lead to death [1]. One of the main factors that contributes to aging is the accumulation of free radicals in the body [2,3]. Free radicals are unstable atoms that steal electrons from other molecules and cause damage to cells and DNA. The body has a natural antioxidant system that can neutralize these free radicals and maintain a balance between oxidation and antioxidants. However, this balance can be disturbed by prolonged exposure to harmful factors such as alcohol, smoking, UV light, or an unhealthy diet [4]. These factors increase the production of free radicals and overwhelm the antioxidant system, leading to oxidative stress and accelerated aging.

Oxidative stress is defined as an imbalance between the production and elimination of reactive oxygen species (ROS), leading to impairment of damage repair by tissue. ROS are highly reactive molecules that can interact with various cellular components, impair their functions, and thus induce cellular senescence [5,6]. Therefore, antioxidant and anti-senescence effects are considered to be important strategies for delaying aging and preventing age-related diseases. Antioxidants are a kind of natural compound that counteracts the effects of oxidation in the body and protects the body from damage [7]. Some antioxidants are produced by the body, while others are obtained from foods. Fruits, vegetables, and other plant-based foods are rich in natural antioxidants, such as vitamin C, vitamin E, carotenoids, anthocyanins, and polysaccharides. These natural antioxidants have been shown to have better antioxidant performance than synthetic ones that are artificially produced [8]. Among the various plant sources of antioxidants, berries and fruits have received special attention for their health benefits. They contain high levels of antioxidant compounds that can enhance human health and prevent or treat various diseases. One example of a berry with remarkable nutritional and medicinal properties is wolfberry (*Lycium barbarum* L. and *L. chinense* Miller.), also known as goji berry [9].

Anthocyanins are polyphenols widely found in berries, vegetables, and other natural plants [10]. They give red, purple, and blue colors to many fruits, vegetables, and other plants. They have various health benefits due to their abilities to modulate different biological processes, such as inflammation, oxidative stress, and cell signaling [11,12,13]. Moreover, anthocyanins have shown a potential to prevent or treat cognitive disorders related to aging, memory impairment, and neuroinflammation [14]. Spermidines are another natural compound that belongs to the polyamine family. They are found in many plants and animals and have multiple functions in the body. Spermidines can protect the cells from damage and stress by inducing autophagy, a process that removes damaged or unnecessary cellular components. Spermidines also have activities against oxidative stress and inflammation. Moderate consumption of spermidine may delay the onset of aging-related diseases [15,16].

*Lycium* L. is a genus that belongs to the Solanaceae family and has a total of 80 species. These species are found worldwide, mainly in Europe and eastern Asia. In China, there are 7 species and 3 varieties of *Lycium* plants that grow in different regions of the country. Three of these species (*L. barbarum* L., *L. ruthenicum*, and *L. chinense* Mill.) are used in the pharmaceutical and food industries [17]. *L. ruthenicum* is a perennial shrub endemic to northwest China, and it grows in arid and semi-arid areas. *L. ruthenicum* has been extensively studied for its potent antioxidants and other bioactive substances that can provide various health benefits [18]. *L. ruthenicum* has been used as a functional food and traditional medicine in China for hundreds of years. The efficacy of *L. ruthenicum* has been documented in many traditional Chinese medical literatures, and it has been verified to have high medicinal values [19]. Recently, the effective activities of *L. ruthenicum* against swelling, inflammation, fatigue, menstrual disorders, cardiovascular disease, cancer, and diabetes have been investigated by various researchers [9,20].

Polysaccharides are the major chemical components of *L. ruthenicum* that have been extensively studied for their health benefits [21]. Modern chemical analysis has confirmed that *L. ruthenicum* is a good source of natural phytochemicals, including anthocyanins and spermidines [9]. Anthocyanins extracted from *L. ruthenicum* have been reported in many studies [22]. Anthocyanin decoration affects stability, bioavailability, and bioactivity. Anthocyanins from *L. ruthenicum* form acylated anthocyanins to increase stability through aromatic acylation [23]. Interestingly, acyl groups enhanced the radical scavenging activity of anthocyanins [24]. We found that anthocyanins and spermidines extracted from *L. ruthenicum* have the same characteristic: they are highly aromatic and possess polarity similarity. They were effectively co-enriched through our extraction process. However, spermidines from *L. ruthenicum* have received less attention. To evaluate the antioxidant activities of these compounds, which are highly aromatic, we obtained a crude extract of *L. ruthenicum* Murr. (LRM) without polysaccharide by using the Liquid Chromatography-Mass Spectrometry (LC-MS) screening method. We then tested the anti-aging effects of the extract in a mouse model of accelerated aging induced by D-galactose injection. D-galactose is a reducing sugar that can cause oxidative stress and cellular senescence in various tissues and organs. We also examined the antioxidant and anti-senescence mechanisms of the extract in Human Ovarian Cancer Cells (A2780) exposed to paraquat (PQ). This study explores the molecular basis of LRMs anti-aging properties and highlights the potential of LRM, which has aromatic compounds, as a functional food and a natural medicine.

## 2. Results

### 2.1. Phytochemical Analysis of LRM

**Figure 1:**
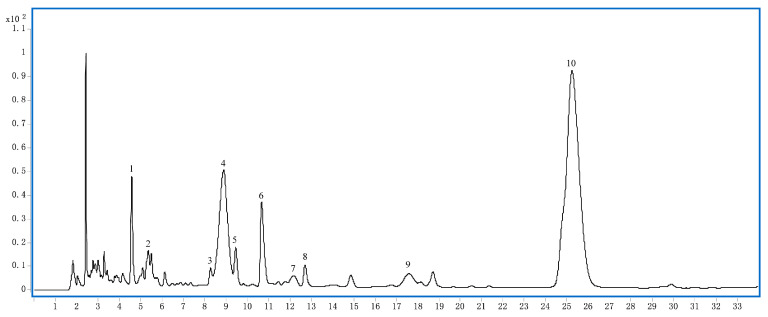
HPLC chromatogram of LRM at 280 nm.

We used HPLC-MS to analyze the phytochemical constituents of LRM. As shown in Figure 1 and Table 1, the main phytochemicals in LRM are anthocyanins and spermidines. Among the anthocyanins, five major compounds accounted for 99% of the total anthocyanin content. The predominant aglycone was petunidin (*m*/*z* 317), followed by delphinidin (*m*/*z* 303), which was identified by MS/MS analysis. The main glycosides were glucosides and rutinosides. Among the spermidines, lycibarbar spermidine B and *N*1-Dihydrocaffeoyl and *N*10-*trans*-caffeoyl-spermidine were the dominant compounds, constituting approximately 24.27% and 39.80% of the total spermidine content, respectively. These results indicate that LRM is rich in anthocyanins and spermidines, which may contribute to its biological activities.

Peak 1 (t_R_ 4.8 min) (Figure S1a, Supplementary Materials) had the molecular ion at *m*/*z* 634 ([M + H]) and five mass fragment ions: *m*/*z* 163, 220, and 310 indicative of the fragmentation of the parent nucleus of N1-*trans*-Caffeoyl, N10-dihydrocaffeoyl spermidine; *m*/*z* 472, corresponding to the loss of one molecule of glucose (Glu); the last one at *m*/*z* 382, corresponding to the Glu link with caffeoyl, but cannot judge glucose connection in meta (Lycibarbar spermidine B) or para (Lycibarbar spermidine D). Based on their MS data and reference [25], peak 1 can be tentatively identified as a pair of Lycibarbar spermidine B&D.

Peak 2 (t_R_ 5.4 min) (Figure S1b, Supplementary Materials) had the molecular ion at *m*/*z* 1081 ([M + H]) and three mass fragment ions: one at *m*/*z* 303, indicative of the fragmentation of one molecule of delphinidin; one at *m*/*z* 919, corresponding to the losses of one molecule of Glu; and the last one at *m*/*z* 465, corresponding to the losses of two molecules of Glu and one molecule of coumalic acid (Cou) and rhamnose (Rha). Based on their MS data and reference [26], peak 2 can be tentatively identified as delphinidin-3-*O*-rutinoside(glucosyl-*trans*-p-coumaroyl)-5-*O*-glucoside.

Peak 3 (t_R_ 8.3 min) (Figure S1c, Supplementary Materials) had the molecular ion at *m*/*z* 474 ([M + H]) and two mass fragment ions: *m*/*z* 165 indicative of the fragmentation of dihydrocaffeoyl and *m*/*z* 222 indicative of the fragmentation of dihydrocaffeoyl linking with NH(CH_2_)_3_. Based on their MS data and reference [25], peak 3 can be tentatively identified as N1-N10-dihydrocaffeoyl spermidine.

Peak 4 (t_R_ 8.8 min) (Figure S1d, Supplementary Materials) and 7 (t_R_ 12.1 min) (Figure S1g, Supplementary Materials) had the same molecular ion at *m*/*z* 1095 ([M + H]) and three mass fragment ions: one at *m*/*z* 317, indicative of the fragmentation of one molecule of petunidin; one at *m*/*z* 933, corresponding to the loss of one molecule of Glu; after that, the last one at *m*/*z* 479, corresponding to the losses of one molecule of Cou, Rha, and Glu. Based on their MS data and reference [26], peak 4 and 7 can be tentatively identified as a pair of petunidin-3-O-rutinoside(glucosyl-*cis*-p-coumaroyl)-5-*O*-glucoside and petunidin-3-*O*-rutinoside(glucosyl-*trans*-p-coumaroyl)-5-*O*-glucoside respectively.

Peak 5 (t_R_ 9.5 min) (Figure S1e, Supplementary Materials) had the molecular ion at *m*/*z* 472 ([M + H]) and three mass fragment ions: *m*/z 163 indicative of the fragmentation of Caffeoyl; *m/z* 220 indicative of the fragmentation of caffeoyl linking with NH(CH_2_)_3_; and one at *m/z* 310, indicative of the fragmentation of one molecule of caffeic acid. Based on their MS data and reference [24], peak 5 can be tentatively identified as N1-*trans*-caffeoyl and N10-dihydrocaffeoyl spermidine.

Peak 6 (t_R_ 10.7 min) (Figure S1f, Supplementary Materials) had the molecular ion at *m*/*z* 472 ([M + H]) and three mass fragment ions: *m*/*z* 163 indicative of the fragmentation of Caffeoyl; *m/z* 222 indicative of the fragmentation of dihydrocaffeoyl linking with NH(CH_2_)_3_; and one at *m/z* 293, indicative of the fragmentation of one molecule of caffeiamide. Based on their MS data and reference [24], peak 6 can be tentatively identified as N1-Dihydrocaffeoyl and N10-*trans*-caffeoyl -spermidine.

Peak 8 (t_R_ 12.7 min) (Figure S1h, Supplementary Materials) had the molecular ion at *m*/*z* 470 ([M + H]) and three mass fragment ions: *m*/*z* 163 indicative of the fragmentation of Caffeoyl; *m*/*z* 220 indicative of the fragmentation of caffeoyl linking with NH(CH_2_)_3_; and one at *m*/*z* 308, indicative of the fragmentation of one molecule of caffeic acid. Based on their MS data and reference [25], peak 8 can be tentatively identified as N1-N10-dihydrocaffeoyl spermidine.

Peak 9 (t_R_ 17.6 min) (Figure S1i, Supplementary Materials) had the molecular ion at *m*/*z* 919 ([M + H]) and three mass fragment ions: one at *m*/*z* 303, indicative of the fragmentation of one molecule of delphinidin; one at *m*/*z* 757, corresponding to the loss of one molecule of glucose Glu; and the last one at *m*/*z* 465, corresponding to the losses of one molecule of Cou, Rha, and Glu. Based on their MS data and reference [26], peak 9 can be tentatively identified as delphinidin-3-*O*-rutinoside(glucosyl-*trans*-p-coumaroyl)-5-*O*-glucoside.

Peak 10 (t_R_ 25.2 min) (Figure S1j, Supplementary Materials) had the molecular ion at *m*/*z* 933 ([M + H]) and three mass fragment ions: one at *m*/*z* 317, indicative of the fragmentation of one molecule of delphinidin; one at *m*/*z* 771, corresponding to the loss of one molecule of glucose Glu; and the last one at *m*/*z* 479, corresponding to the losses of one molecule of Cou, Rha, and Glu. Based on their MS data and reference [26], peak 9 can be tentatively identified as etunidin-3-O-rutinoside (*trans*-p-coumaroyl)-5-*O*-glucoside.

### 2.2. Effect of LRM Pretreatment on D-Gal-Induced Cognitive Ability in Mice

**Figure 2:**
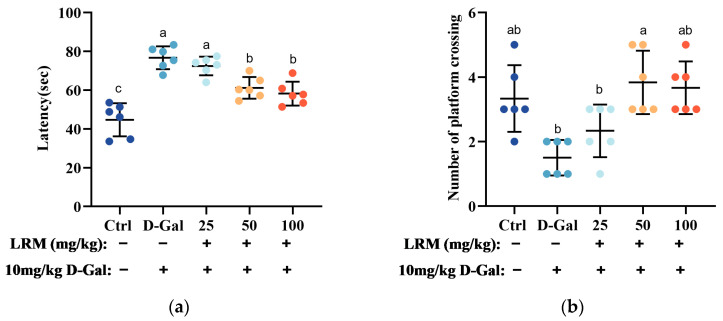
Effect of LRM pretreatment on D-galactose (D-Gal)-induced cognitive ability in mice. (**a**) Latency in removing the platform after the training section. (**b**) The number of platform crossings during the test of the Morris water maze (MWM). Data were presented as means ± SD (*n* = 6). Values with different letters in each column were significantly different (*p* < 0.05).

Morris water maze (MWM) is a widely used behavioral test to assess the spatial learning and memory of rodents [27]. In this test, mice are trained to find a hidden platform in a circular pool using distal cues. The escape latency (the time to find the platform) and the number of platform crossings (the frequency of crossing the platform location during a probe trial) are used as indicators of spatial learning and memory. In this study, we performed the MWM test to evaluate the effects of LRM treatment on the cognitive function of mice treated with D-galactose (D-Gal). As shown in Figure 2, mice in the D-Gal group showed longer escape latencies than those in the control group, suggesting impaired spatial learning abilities in mice. However, mice in the LRM groups showed significantly shorter escape latencies than mice treated with D-Gal, suggesting improved spatial learning abilities in mice. The probe trial results showed that D-Gal decreased the number of platform crossings, suggesting impaired spatial memory abilities in mice. However, LRM treatment increased the number of platform crossings in D-Gal-treated mice, suggesting improved spatial memory abilities in mice. These results indicated that LRM alleviated cognitive impairment in mice caused by D-Gal by enhancing their ability to acquire and retain spatial information.

### 2.3. Effect of LRM Treatment on Oxidative/Antioxidant Status in Mice

**Figure 3:**
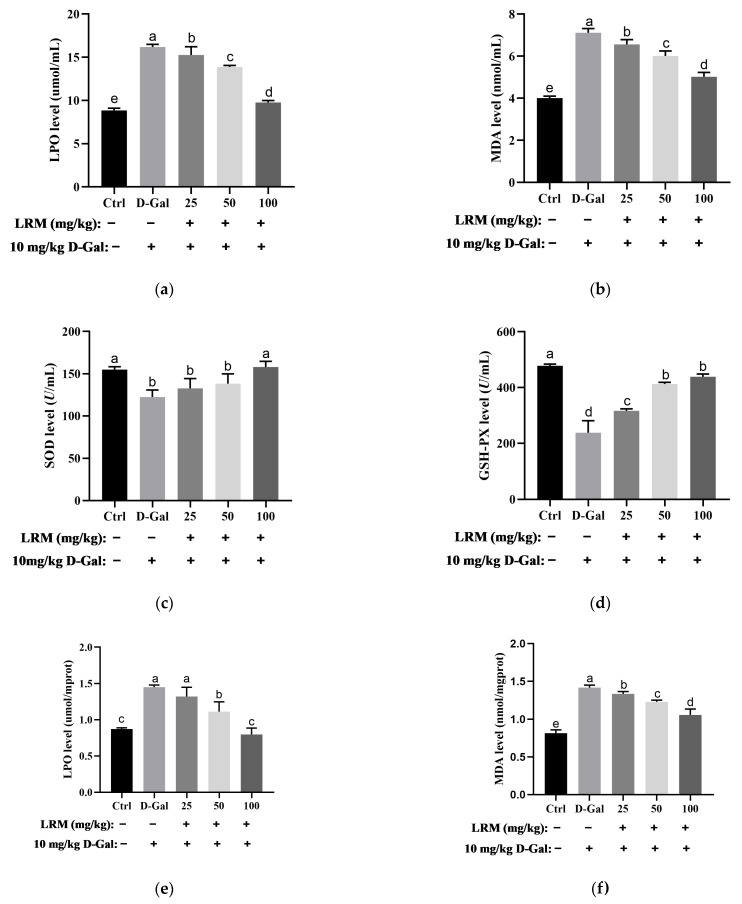

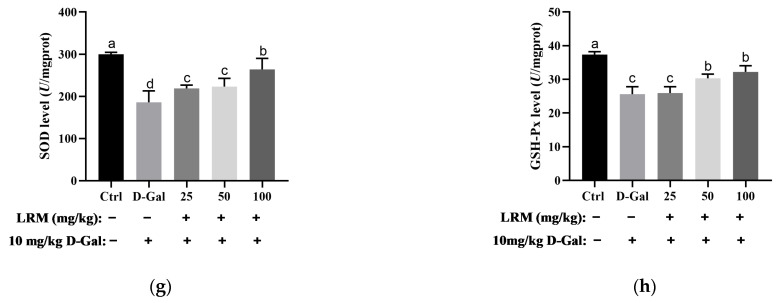
Effect of LRM treatment on oxidative/antioxidant status in mice (*n* = 6 per group). Serous levels of (**a**) lactoperoxidase (LPO), (**b**) malondialdehyde (MDA), and activities of (**c**) superoxide dismutase (SOD), and (**d**) glutathione peroxidase (GSH-Px). Contents of (**e**) LPO and (**f**) MDA, and the enzyme activities of (**g**) SOD and (**h**) GSH-Px in the brains of mice. Values with different letters in each column were significantly different (*p* < 0.05).

To evaluate the effects of LRM treatment on the antioxidant status of mice, we measured the lipid peroxidation product levels and antioxidant enzyme activities in the serum after D-Gal and LRM treatment. Lipid peroxidation products, such as lipid peroxidation (LPO) and malondialdehyde (MDA), reflect the extent of oxidative damage to cell membranes. As shown in Figure 3, D-Gal treatment significantly increased the levels of LPO and MDA in the serum, suggesting enhanced lipid peroxidation in mice. However, LRM treatment markedly reduced the contents of LPO and MDA in D-Gal-treated mice, indicating that LRM could protect cell membranes from oxidative damage.

Antioxidant enzymes, such as superoxide dismutase (SOD) and glutathione peroxidase (GSH-Px), play a crucial role in scavenging ROS and maintaining redox balance. SOD catalyzes the dismutation of O_2_^•−^ to H_2_O_2_, while GSH-Px reduces H_2_O_2_ to water using glutathione as a cofactor [28,29]. D-Gal treatment significantly decreased the enzyme activities of SOD and GSH-Px in the serum, suggesting impaired antioxidant defense abilities in mice. However, LRM treatment significantly increased the levels of SOD and GSH-Px in D-Gal-treated mice, suggesting enhanced antioxidant defense abilities in mice.

In addition to the serum, we also measured the levels of peroxides and antioxidant enzymes in the brain tissue, which can reflect the levels of oxidative stress in mice. Peroxide and antioxidant enzyme levels in mouse brain tissue showed consistent trends in serum. The contents of LPO and MDA in the brain tissue of D-Gal-treated mice were significantly higher than those in the control group. However, the contents of LPO and MDA decreased significantly after LRM gavage. SOD and GSH-Px enzyme activities decreased significantly in the D-Gal-treated group. However, they were significantly increased in the LRM-treated group, which showed a dose-dependent effect. These results highlighted that LRM could attenuate oxidative stress in mice by modulating both ROS production and elimination.

### 2.4. Effects of LRM Pretreatment on A2780 Cellular Senescence Induced by PQ

**Figure 4:**
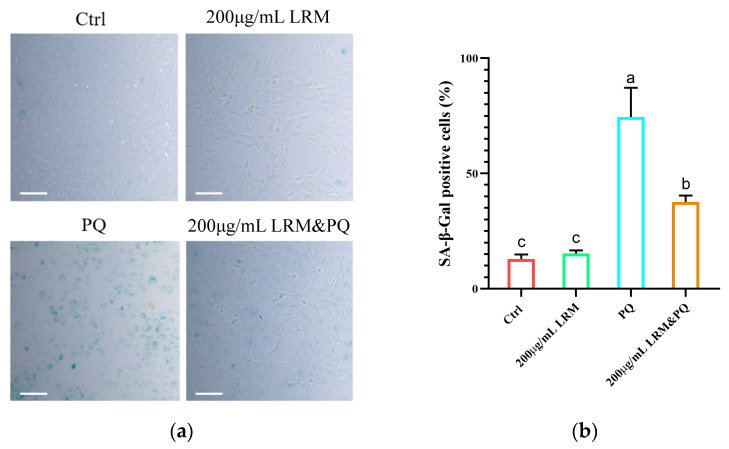
Effects of LRM pretreatment on A2780 cellular senescence induced by paraquat (PQ). (**a**) Micrographs of cell senescence detected by β-galactosidase staining in Ctrl, 200 µg/mL LRM treatment, 200 µg/mL PQ treatment, and the 200 µg/mL LRM and 200 µg/mL PQ cotreatment groups after 24 h of treatment. The scale bar represents 200 μm. (**b**) Statistical plots of the proportion of senescent cells (**a**). Values with different letters in each column were significantly different (*p* < 0.05).

PQ can induce cellular senescence. In this study, we measured the senescence rate of A2780 cells (labeled with a senescence β-galactosidase staining kit) after PQ treatment. Compared with normal cells, senescent cells are usually larger and express a highly active β-galactosidase that can react with X-Gal to produce a dark blue substrate. After 24 h, high-concentration PQ (200 µg/mL) treatment significantly induced cellular senescence. However, when A2780 cells were pretreated with 200 µg/mL LRM and 200 µg/mL PQ, the rate of cellular senescence was significantly suppressed compared with the group treated with PQ alone (Figure 4). This result indicated that LRM could protect A2780 cells from PQ-induced cellular senescence.

### 2.5. Effects of LRM Pretreatment on ROS Production in A2780 Cells Induced by PQ

**Figure 5:**
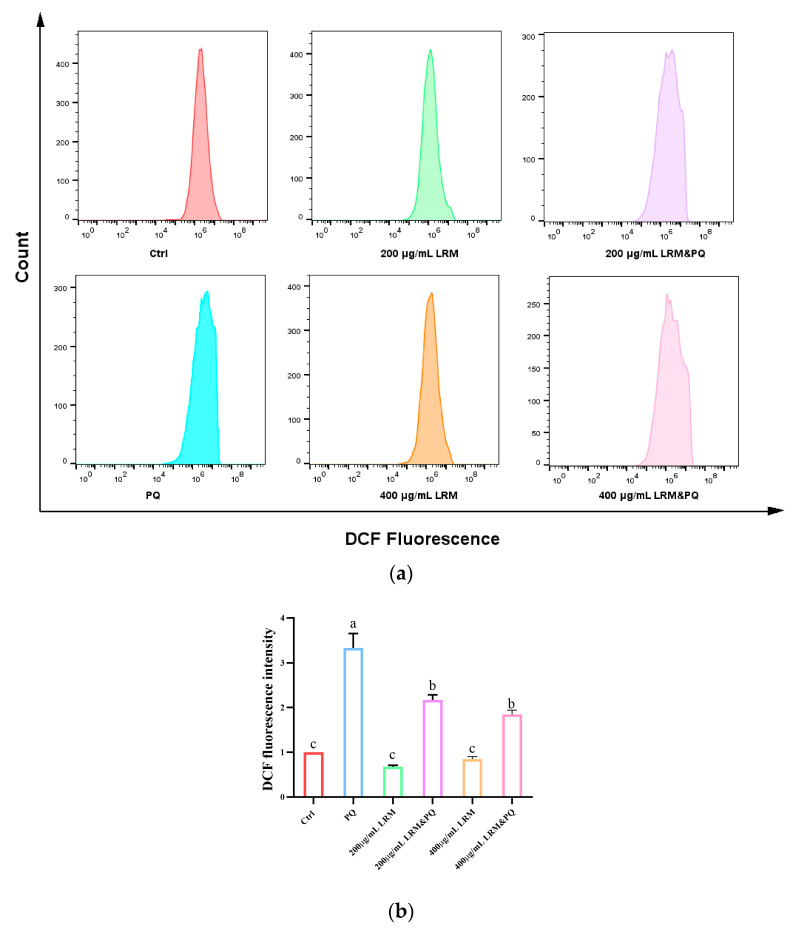
Effects of LRM pretreatment on ROS production in A2780 cells induced by PQ. (**a**) A total of 2′,7′-Dichlorodihydrofluorescein (DCF) fluorescence intensity of A2780 cells in Ctrl, 200 µg/mL LRM treatment, 400 µg/mL LRM treatment, 200 µg/mL PQ treatment, 200 µg/mL LRM and 200 µg/mL PQ cotreatment, and 400 µg/mL LRM and 200 µg/mL PQ cotreatment group after 24 h of treatment. (**b**) Bar graph of DCF fluorescence intensity as presented in (**a**). Values with different letters in each column were significantly different (*p* < 0.05).

A high level of oxidative stress contributes to biomolecular damage and cell death through the mitochondrial apoptosis pathway. ROS are a kind of signaling molecule at physiological levels that participate in various cellular processes, including proliferation, differentiation, programmed cell death, autophagy, redox signaling, and hypoxic stress response [30]. Approximately 2′,7′-Dichlorodihydrofluorescein diacetate (DCFH-DA) can be hydrolyzed to 2′,7′-Dichlorodihydrofluorescein (DCFH) by esterase, and DCFH can be oxidized by intracellular ROS to 2′,7′-Dichlorodihydrofluorescein (DCF), which has green fluorescence. After 24 h of treatment, compared with the control group, the intracellular ROS level was decreased in the 200 µg/mL or 400 µg/mL LRM treatment group, and it increased after 200 µg/mL PQ treatment. When 200 µg/mL or 400 µg/mL LRM and 200 µg/mL PQ are applied together, intracellular ROS are at low levels (Figure 5). Thus, LRM could significantly decrease the intracellular ROS level induced by PQ, thereby alleviating the intracellular oxidation level.

### 2.6. Effects of LRM Pretreatment on Mitochondrial Membrane Potential (MMP) of A2780 Induced by PQ

**Figure 6:**
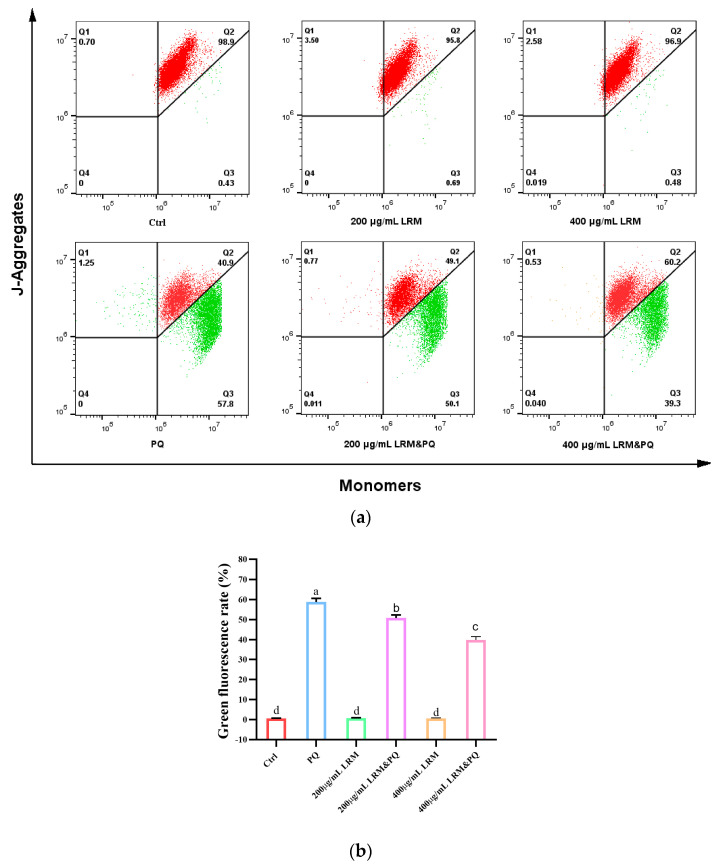
Effects of LRM pretreatment on MMP in A2780 cells induced by PQ. (**a**) A total of 5,5′,6,6′-Tetrachloro-1,1′,3,3′-tetraethylbenzimidazolylcarbocyanine iodide (JC-1) fluorescence intensity of A2780 cells in Ctrl, 200 µg/mL LRM treatment, 400 µg/mL LRM treatment, 200 µg/mL PQ treatment, 200 µg/mL LRM and 200 µg/mL PQ cotreatment, and 400 µg/mL LRM and 200 µg/mL PQ cotreatment group after 24 h of treatment. The encompassed population of Q2 represents J-aggregates, whereas Q3 represents monomers. (**b**) Bar graph of JC-1 fluorescence intensity as presented in (**a**). Values with different letters in each column were significantly different (*p* < 0.05).

High levels of ROS can reduce MMP and promote cellular senescence. Approximately 5,5′,6,6′-Tetrachloro-1,1′,3,3′-tetraethylbenzimidazolylcarbocyanine iodide (JC-1) can selectively cross the mitochondrial membrane and change its fluorescence characteristics. When cells are healthy, the MMP is high and JC-1 forms a polymer that emits red fluorescence; when cells are senescent, the MMP is low and JC-1 exists as a monomer that emits green fluorescence. Compared with the control group, the fluorescence intensity was not significantly different after being treated with 200 µg/mL or 400 µg/mL LRM for 24 h. When treated with 200 µg/mL PQ for 24 h, the red fluorescence was weakened and the green fluorescence was significantly enhanced, indicating that PQ treatment can significantly reduce the MMP level. However, when treated with 200 µg/mL or 400 µg/mL LRM and 200 µg/mL PQ, the green fluorescence was significantly weakened (Figure 6), indicating that LRM treatment can significantly suppress the reduction of MMP induced by PQ.

## 3. Discussion

LRM is a natural product derived from *L. ruthenicum*, a plant that has been shown to have beneficial effects against oxidative stress and age-related disorders such as diabetes, cardiovascular diseases, neurodegeneration, and cancer [9,20]. LRM contains various anthocyanins and spermidines, which are potential anti-aging agents. Anthocyanins are natural pigments that have potent anti-oxidant and anti-inflammatory activities [31], while spermidines are polyamines that can modulate autophagy and cellular senescence [32]. Anthocyanins and spermidines extracted from *L. ruthenicum* have the same characteristic: they are highly aromatic and possess polarity similarity, which makes them effectively co-enriched. In this study, we used LC-MS analysis to identify 5 anthocyanins and 5 spermidines in LRM. We found that the anthocyanins in LRM had two different aglycones: petunidin and delphinidin. These compounds might have had different biological activities and mechanisms of action. We performed in vivo and in vitro experiments to investigate the anti-aging effects of LRM and its constituents. We evaluated the protective effects of LRM against memory impairment and oxidative stress induced by D-Gal in mice. We also examined the anti-senescence effects of LRM against PQ-treated A2780 cells. Our results may provide new insights into the pharmacological properties of LRM in aging-related disorders.

Aging is the main cause of a gradual decline in brain function, usually accompanied by cognitive impairment, memory loss, and dementia [33]. To promote healthy aging and prevent age-related health problems, effective anti-aging interventions need to be developed. One of the possible causes of aging is oxidative stress [34], which is often associated with age and age-related neurodegenerative diseases such as Alzheimer’s and Parkinson’s disease [35]. These diseases cause cognitive decline and neuronal loss, and the severity of cognitive impairment is correlated with the degree of oxidative stress [36]. Oxidative stress occurs when free radicals such as ROS accumulate excessively in the body due to harmful stimuli and disrupt the dynamic balance between oxidation and anti-oxidation. This leads to decreased SOD activity and/or increased MDA content, abnormal activation of signal transduction pathways, induction of apoptosis, and tissue damage. The brain and nervous system are prone to oxidative stress and lack sufficient antioxidant defense systems to prevent oxidative damage [37]. For example, AD patients have reduced the enzyme activities of SOD and GSH-Px and the content of MDA in their serum, indicating the presence of oxidative stress and free radical damage [38]. These suggested that oxidative stress might play an important role in the process of cognitive impairment. Our study results showed that after 6 weeks of long-term injection of D-Gal, mice have increased the contents of LPO and MDA in serum and brain and decreased the enzyme activities of the antioxidant enzymes GSH-Px and SOD, indicating that oxidative-antioxidant balance is disrupted, which was consistent with previous studies. Therefore, regulating the production of free radicals or reducing their harmful effects can be used as a potential therapeutic strategy for preventing and controlling aging and cognitive impairment [39]. Previous studies have shown that some natural compounds, such as quercetin, can improve learning memory and cognitive memory in animal models of neurodegenerative diseases by reducing oxidative stress and mitochondrial dysfunction [40]. In this study, we found that LRM had a similar effect. LRM successfully reduced the time for mice to find the platform and increased the number of times they crossed the platform in a water maze test, indicating that it can improve the cognitive abilities of mice. Moreover, LRM reduced the levels of LPO and MDA in serum and brain and increased the enzyme activities of SOD and GSH-Px, indicating it can enhance the antioxidant capacity of mice. These study results highlighted that LRM has the potential pharmacological effect of improving cognitive ability by reducing oxidative stress.

Cellular senescence is a response elicited by aging [41]. Pharmacological elimination of senescent cells could extend the health span and longevity of natural aging mice [42]. Cellular senescence is also an important response to oxidative stress and mitochondrial damage. Mitochondria plays an important role in the progressively declining abilities of energy provision and redox homeostasis during aging [43]. Oxidative stress is caused by an imbalance between the production and elimination of ROS, which can damage cellular components and induce aging [44,45]. Meanwhile, there is a direct causal relationship between oxidative stress and mitochondrial dysfunction, and oxidative stress-mediated mitochondrial dysfunction facilitates cell senescence [46]. Chen Qing found that PQ induced expression of cellular senescence marker P21 and mitochondrial ROS production, thus facilitating cellular senescence [47,48]. Benjamin discussed oxidative stress, mitochondrial dysfunction, anti-stress ability, and longevity in a PQ-treated *C. elegans* model [49]. Thus, using PQ as a redox cycler to stimulate superoxide production, we here discussed the effect of LRM on PQ-induced aging in A2780 cells and mitochondrial dysfunction induced by ROS. We found that LRM did not affect cell viability at the tested concentrations, indicating that it was not cytotoxic to A2780 cells. LRM significantly reduced the senescence rate and intracellular ROS level and suppressed the reduction of MMP in A2780 cells induced by PQ treatment. In other studies, the PQ model was widely used to screen natural products for anti-senescence activities. Huang found that Ginsenoside Rg1 protected pulmonary epithelial cells from PQ-induced cellular senescence in an ATG12-dependent manner [50]. Berberine significantly decreased PQ-induced cell death, ROS formation, and MMP reduction in primary cultured rat hepatocytes [51]. These results highlighted that LRM could protect A2780 cells from PQ-induced cellular senescence. Our results demonstrated that LRM had potential anti-senescence activity on A2780 cells through reducing ROS accumulation and protecting mitochondrial functions.

Aging is a complex and multifactorial process that involves progressive physiological damage to genes, cells, tissues, and the entire organism, which ultimately affects the functional efficiency of organs [52]. Oxidative stress is one of the major factors that contributes to aging and age-related diseases, as it causes damage to biomolecules and cellular structures [53]. In vitro and in vivo experiments have verified that LRM inhibits aging by inhibiting ROS accumulation. In in vitro experiments, LRM inhibits cellular senescence by scavenging ROS and protecting mitochondria. In in vivo experiments, LRM increased the levels of antioxidant enzymes, such as SOD and GSH-Px, and protected cell membranes from oxidative damage. Therefore, antioxidant effects are important strategies for delaying aging and preventing age-related diseases.

Our results indicated that LRM had multiple effects on anti-aging and antioxidation in vitro and in vivo. However, we acknowledge that our results are not conclusive and that there may be other mechanisms involved in the anti-aging activity of LRM. For example, LRM may modulate other signaling pathways or transcription factors related to aging, such as AMPK, SIRT1, Nrf2, NF-κB, etc. Moreover, anthocyanins and spermidines may have synergistic or additive effects with other components of LRM, such as polysaccharides, flavonoids, phenolic acids, etc. Therefore, further studies are needed to elucidate the detailed molecular mechanisms and interactions between LRM and the prevention of age-related diseases.

## 4. Materials and Methods

### 4.1. Reagents

Ethanol was purchased from Tianjin Saifurui Technology Company (Tianjin, China). Acetonitrile and formic acid were obtained from Tedia Company (Fairfield, IA USA). D-Galactose (D-Gal) was purchased from Yuanye Bio-Technology Company (Shanghai, China). Chloral hydrate was purchased from Ruichengkang Pharmaceutical Technology (Shanxi) Company (Xi’an, Shanxi, China). Total Superoxide Dismutase (T-SOD) assay kit (Hydroxylamine method), Glutathione Peroxidase (GSH-Px) assay kit, Malondialdehyde (MDA) assay kit (TBA method), and Lipid Peroxidation (LPO) assay kit were obtained from Nanjing Jiancheng Bioengineering Institute (Nanjing, Jiangsu,, China). Dulbecco’s Modified Eagle Medium (DMEM) and penicillin/streptomycin were purchased from Gibco Company (Grand Island, NY, USA), and Fetal Bovine Serum (FBS) was purchased from Anhui White Shark Biotechnology Company (Hefei, Anhui, China). Phosphate buffered saline (PBS) was purchased from Abixin (Shanghai) Biotechnology Company (Shanghai, China). H_2_O_2_ was purchased from Sigma-Aldrich Company (Darmstadt, Germany). Paraquat was purchased from Macklin Biochemical Technology Company (Shanghai, China). Senescence β-Galactosidase staining kit and JC-1 were purchased from Beyotime Biotech Company (Shanghai, China). DCFH-DA was purchased from Yuanye Biotechnology Company (Shanghai, China).

### 4.2. Plant Materials

*L. ruthenicum* (heavy metal < 20 ppm, pesticide residues < 2 ppm) was obtained from the barbary wolfberry planting base of the Barbary Wolfberry Engineering Technology Research Institute, Ningxia Academy of Agriculture and Forestry Sciences (Ningxia, China) in August 2022 and identified by Mei Tian (Jiangsu Key Laboratory for the Research and Utilization of Plant Resources, Institute of Botany, Jiangsu Province, and Chinese Academy of Sciences (Nanjing, China)). The dried fruit samples (HGGQG20220723) were stored in the herbarium of the Institute of Botany, Jiangsu Province, and the Chinese Academy of Sciences (Nanjing, China). The dried fruits were extracted with 70% ethanol at 45 °C; the solid-liquid ratio was 1:8 for 2 h, 1:6 for 1 h, and then 1:6 for 1 h. The macerated product was filtered and combined; the extract liquid was applied to an Amberly (1 kg) column and eluted with 4-fold column volumes of 5% formic acid; and the aqueous solution was discarded. Then, the column was eluted with 4-fold column volumes of EtOH-H_2_O-HCOOH (50:50:5), and the eluent was condensed to a density of 1.2 g/cm^3^ and freeze-dried. The extract collected was labeled as *L. ruthenicum* Murr. extract (LRM).

### 4.3. HPLC-DAD Analysis

Twenty milligrams of LRM were dissolved in 2 mL of acetonitrile, water, and formic acid (50:50:1) mixed solution, sonicated in an ultrasonic cleaner to dissolve the powder completely, and filtered through a 0.22 μm membrane for HPLC-DAD analysis. An Agilent 1260 HPLC-DAD-6840 ESI-QTOF MS system equipped with a C_18_ analytical column (4.6 × 100 mm, 2.7 μm, Agilent, Santa Clara, CA, USA) and an ultraviolet−visible (UV−vis) detector was used for analysis. Ten microliters of LRM/acetonitrile/water/formic acid solution were used for analysis. The mobile phase was composed of 1% formic acid in water (A) and acetonitrile (B), and the gradient elution was as follows: 0 min, 10% B; 35 min, 15% B; 40 min, 20% B; 50 min, 100% B. The flow rate of 0.5 mL/min was applied for elution, the column temperature was 40 °C, and UV-detection was performed at 280 nm.

### 4.4. Q-TOF MS Conditions

The mass spectrum detection system includes: Ionization Mode Dual ESI; positive ion scanning mode (1000–3200 *m*/*z*); instrument mode: 4 GHz high resolution mode; nebulizer pressure: 50 psi; drying gas flow: 10 mL·min^−1^; drying gas temp: 350 °C; capillary voltage: 4000 V; fragmentor voltage: 205 V; MS/MS collision energy: 35 V.

### 4.5. Animals and Treatment

Thirty male mice (45 days, 18–23 g) were used in this study. The Kunming mice SPF were purchased from Beijing Vital River Laboratory Animal Technology Co., Ltd. (Beijing, China). Mice were housed under standard experimental conditions for 20 days: humidity (50~60%), temperature (21~25 °C), light (12 h/12 h light/dark cycle), new bedding material was changed every two days, and mice were fed on a standard diet. Experiments were conducted under the guidance of the National Institute of Health on Animal Care Principles.

Mice were randomly divided into five groups (*n* = 6 per group) as follows: treated with 0.9% saline (Control), 100 mg/kg D-galactose (D-Gal group), 100 mg/kg D-galactose plus 25 mg/kg LRM (low dose), 100 mg/kg D-galactose plus 50 mg/kg LRM (middle dose), or 100 mg/kg D-galactose plus 100 mg/kg LRM (high dose). D-galactose was administered by intraperitoneal injection and LRM by intragastric treatment once daily for 6 weeks.

### 4.6. Morris Water Maze (MWM) Test

The cognitive function and memory capacity of mice were evaluated after intraperitoneal injection and intragastric administration using the MWM test [27]. Mice were placed in an opaque circular tank, which is 150 cm in diameter and 50 cm in height, and filled with water maintained at a temperature of 24 ± 1 °C during the test. An escape platform with a diameter of 12 cm was located at the center of the tank and was 1 cm below the water surface. During the 5-day learning phase, the latency to reach the platform was measured for each mouse. On day 6, the platform was removed for a probe test, in which each mouse was allowed to swim freely for 60 s. The latency to cross the platform location for the first time and the number of platform crossings were recorded.

### 4.7. Anesthetization and Sample Collection

After the MWM test, mice in each group were deeply anesthetized with chloral hydrate (400 mg/kg) through intraperitoneal injections, and their blood was collected from the right ventricular. The collected blood was incubated at 37 °C for 1 h and placed in the refrigerator at 4 °C overnight. After centrifugation at 4000 r/min for 15 min, the serum was rapidly frozen in liquid nitrogen and stored at −80 °C for further analysis. The collected brain was washed with normal saline, wrapped in tinfoil, and stored at −80 °C for further analysis.

### 4.8. Determination of the Contents of LPO and MDA and the Enzyme Activities of SOD and GSH-Px

The determination of the contents of LPO and MDA and the enzyme activities of SOD and GSH-Px in serum and brain tissue were vital for the assessment of oxidative stress [54]. The SOD, GSH-Px, MDA, and LPO levels were detected using a series of Assay Kits (Nanjing Jiancheng Bioengineering Institute, Nanjing, China). Oxidative stress biochemical parameters were measured according to the kit manufacturer’s instructions.

The total SOD in serum was detected using a spectrophotometric method. The activity of SOD that corresponds to 50% SOD inhibition per mL of serum is one unit of SOD activity (U). GSH-Px promotes the reaction between H_2_O_2_ and reduced glutathione (GSH) to form H_2_O and oxidized glutathione (GSSG). The activity of GSH-Px (U) can be represented as the rate of enzymatic reaction. One U exhibits that decreasing in GSH concentration by 1 mol/L is one unit of enzyme activity under the method’s condition. MDA content was detected using the method of condensation with thiobarbituric acid, with a maximum absorption peak downstream at 532 nm (a standard curve prepared with 1,1,3,3-tetraethoxypropane). LPO reacts with the chromogenic agent in the commercial kit with spectrophotometric detection using the excitation wavelength of 586 nm.

### 4.9. Cell Culture and Treatment

The human ovarian cancer cell line A2780 was obtained from ATCC and cultured in DMEM medium supplemented with 1% penicillin/streptomycin and 10% heat-inactivated fetal bovine serum. Cells were incubated at 37 °C in a humidified atmosphere of 95% air/5% CO_2_. LRM or PQ was dissolved in DMEM medium, and all cells were pre-treated with four different methods as follows: treated with DMEM medium (Control), 200 µg/mL LRM, 400 µg/mL LRM, 200 µg/mL PQ, 200 µg/mL LRM and 200 µg/mL PQ, or 400 µg/mL LRM and 200 µg/mL PQ for 24 h. All assays were performed in triplicate.

### 4.10. Cellular Senescence Assay

The senescence β-Galactosidase staining kit was used to detect cellular senescence [55]. A2780 cells in the logarithmic growth phase were pretreated as follows: treated with DMEM medium (Control), 200 µg/mL LRM, 200 µg/mL PQ, or 200 µg/mL LRM and 200 µg/mL PQ for 24 h. After washing with PBS, 1 mL of β-Galactosidase fixative solution was added to fix the cells for 15 min at room temperature. The cells were then washed with PBS three times and stained with 1 mL of staining solution for 12 h at 37 °C. After that, these cells were analyzed with the microscope (Olympus IX51, Olympus Corporation, Tokyo, Japan). Image J (Image J 1.53 S, National Institutes of Health, Bethesda, MD, USA) was used to analyze and visualize the results.

### 4.11. Cell ROS Assay

We referred to the method of N. Lin et al. for the analysis of cytosolic ROS levels and made some improvements [56]. A2780 cells in the logarithmic growth phase were pretreated as mentioned in 4.9. The cells were treated with trypsin without EDTA. After centrifugation at 1000× *g* for 5 min, the cells were washed with PBS. The cells were resuspended in serum-free medium containing a final concentration of 20 µM DCFH-DA. After incubation for 20 min at 37 °C and washing with PBS, these cells were examined using a flow cytometer (BD Accuri C6, BD Biosciences, San Jose, CA, USA). Flow Jo v10.0.7 (BD Biosciences, San Jose, CA, USA) was used to analyze and visualize the results.

### 4.12. Mitochondrial Transmembrane Potential Assay

We referred to the method of Manna A. et al. for the analysis of MMP levels and made some improvements [57]. A2780 cells in the logarithmic growth phase were pretreated as mentioned in 4.9. The cells were treated with trypsin without EDTA. After centrifugation at 1000× *g* for 5 min, the cells were washed with PBS. The cells were resuspended in serum-free medium containing a final concentration of 20 µg/mL JC-1. After incubation for 20 min at 37 °C and washing with PBS, these cells were examined using a flow cytometer (BD Accuri C6 Plus, BD Biosciences, San Jose, CA, USA). Flow Jo v10.0.7 (Flow Jo, LLC) was used to analyze and visualize the results.

### 4.13. Statistical Analysis and Other Tests

GraphPad Prism 9.0.0 (GraphPad Prism, La Jolla, CA, USA) was used to statistically evaluate all data. Intergroup comparisons were determined by one-way ANOVA and Tukey’s multiple comparisons test. All experimental data were presented as the mean ± standard deviation (SD). A statistical difference was considered significant if *p* < 0.05.

## 5. Conclusions

*L. ruthenicum* is a medicinal plant that contains high levels of anthocyanins and spermidines, which have antioxidant and anti-aging effects. In this study, we evaluated the effects of LRM on memory impairment and oxidative stress induced by D-Gal in mice. We also tested the anti-senescence activity of LRM in A2780 cells exposed to PQ. We found that LRM improved the abilities of cognitive function and spatial memory in D-Gal-treated mice by reducing oxidative stress. LRM also protected A2780 cells from PQ-induced mitochondrial damage and cellular senescence by scavenging ROS levels. Our results suggest that LRM has multiple effects on aging, which may be mediated by its active components. LRM may have the potential to be a functional food or a healthcare product for the treatment of aging and age-related diseases.

## Figures and Tables

**Table 1:** Anthocyanins and spermidines were identified by HPLC-Triple-TOF/MS.

No.	Compound	t_R_(min)	[M + H]^+^	MS/MS [*m*/*z*]
1	Lycibarbar spermidine B&D	4.8	634.2930	163/220/310/ 382/472/634/
2	Delphinidin-3-*O*-rutinoside(glucosyl-*trans*-p-coumaroyl)-5-*O*-glucoside	5.4	1081.2907	303/465/919/ 1081
3	N1, N10-dihydrocaffeoyl spermidine	8.3	474.2595	165/222/474
4	Petunidin-3-*O*-rutinoside(glucosyl-*cis*-p-coumaroyl)-5-*O*-glucoside	8.8	1095.3116	317/479/933/ 1095
5	N1-trans-Caffeoyl, N10-dihydrocaffeoyl spermidine	9.5	472.2434	163/220/310/ 472
6	N1-Dihydrocaffeoyl, N10-*trans*-caffeoyl -spermidine	10.7	472.2436	163/222/293 472
7	Petunidin-3-*O*-rutinoside(glucosyl-*trans*-p-coumaroyl)-5-*O*-glucoside	12.1	1095.3112	317/479/933/ 1095
8	N1, N10-dicaffeoyl-spermidine	12.7	470.2267	163/220/308/ 470
9	Delphinidin-3-*O*-rutinoside (*trans*-p-coumaroyl)-5-*O*-glucoside	17.6	919.2449	303/465/757/ 919
10	Petunidin-3-*O*-rutinoside (*trans*-p-coumaroyl)-5-*O*-glucoside	25.2	933.2616	317/479/771/ 933

## Data Availability

Research data are not shared.

## Supplementary Materials

The following supporting information can be downloaded at: https://www.mdpi.com/article/10.3390/molecules28227615/s1, Figure S1: Secondary mass spectrum.
