# Supplementary material for: The Ethanolic Extract of Lycium ruthenicum Ameliorates Age-Related Physiological Damage in Mice
Source: Molecules. 2023 Nov 15;28(22):7615. doi: 10.3390/molecules28227615 (PMC10673502; doi:10.3390/molecules28227615)
Supplement: Supplementary file 1 [file molecules-28-07615-s001.zip › molecules-2622775-supplementary.pdf]

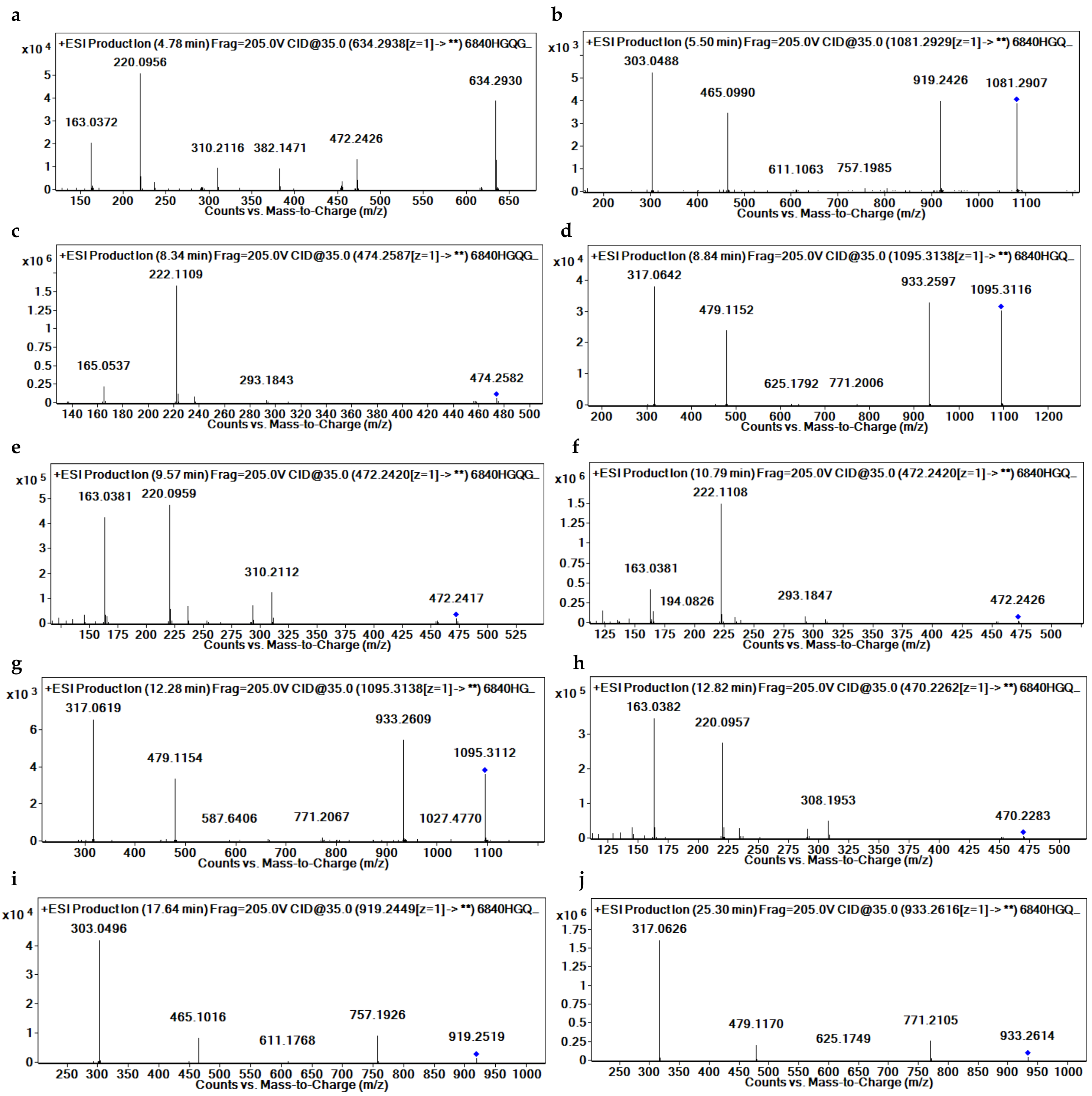

Figure S1. Secondary mass spectrum **(a)** Secondary mass spectrum of Lycibarbar spermidine B&D, **(b)** Secondary mass spectrum of Delphinidin-3-O-rutinoside(glucosyl-trans-p-coumaroyl)-5-O-glucoside, **(c)** Secondary mass spectrum of N1, N10-dihydrocaffeoyl spermidine, **(d)** Secondary mass spectrum of Petunidin-3-O-rutinoside(glucosyl-cis-p-coumaroyl)-5-O-glucoside, **(e)** Secondary mass spectrum of N1-trans-Caffeoyl, N10-dihydrocaffeoyl spermidine, **(f)** Secondary mass spectrum of N1-Dihydrocaffeoyl, N10-*trans*-caffeoyl -spermidine, **(g)** Secondary mass spectrum of Petunidin-3-O-rutinoside(glucosyl-*trans*-p-coumaroyl)-5-O-glucoside, **(h)** Secondary mass spectrum of N1, N10-dicaffeoyl-spermidine, **(i)** Secondary mass spectrum of Delphinidin-3-O-rutinoside (*trans*-p-coumaroyl)-5-O-glucoside, **(j)** Secondary mass spectrum of Petunidin-3-O-rutinoside (*trans*-p-coumaroyl)-5-O-glucoside.
